# User Requirements and Conceptual Design for an Electronic Data Platform for Interhospital Transfer Between Acute Care Hospitals: User-Centered Design Study

**DOI:** 10.2196/67884

**Published:** 2025-05-30

**Authors:** Pamela Garabedian, Jazzarae Kain, Srinivas Emani, Stephanie Singleton, Ronen Rozenblum, Lipika Samal, Stephanie Mueller

**Affiliations:** 1 Research Information Science & Computing, Mass General Brigham, 399 Revolution Drive, Somerville, MA, 02145, United States; 2 Division of General Internal Medicine, Brigham and Women's Hospital, Boston, MA, United States; 3 Harvard Medical School, Boston, MA, United States

**Keywords:** interhospital transfer, health information exchange, user-centered design, electronic health record, electronic data, acute care, conceptual design, health information, user requirement, qualitative, data access, clinical data, design, user design, decision-making, decision support

## Abstract

**Background:**

The transfer of patients between hospitals, that is, interhospital transfer (IHT), introduces discontinuity of care, including gaps in health information transfer, which may worsen patient outcomes.

**Objective:**

This is the first phase of a 5-year research study. Our goals are (1) to understand the gaps in health information exchange (HIE) and the clinician experience in accessing and using the electronic health record (EHR) during IHT and (2) to identify clinician user requirements for the development of an internal EHR solution for IHT.

**Methods:**

We used prior work on HIE during IHT, coupled with a user-centered design (UCD) process to engage in discussions with clinical users and gather input on EHR workflow during IHT patient admission and planning. A total of 8 UCD sessions were held between February and July 2023, involving 18 clinicians who interact with the EHR during IHT, including 3 medicine residents, 10 advanced practice providers (APPs), and 5 direct care attendings—all responsible for caring for IHT patients at Brigham and Women’s Hospital Cardiology, Medicine, Oncology, and intensive care unit services. Discussions highlighted facilitators and barriers and suggested improvements for data access and availability at the time of transfer. UCD sessions were recorded, analyzed, and coded by 2 independent reviewers to identify common themes driving suboptimal HIE. User requirements were derived from the sessions with users and iteratively refined throughout the process.

**Results:**

Qualitative analysis revealed that a significant number of frontline clinicians experience suboptimal availability of clinical information in the EHR at the time of IHT, including gaps in communication, incomplete data, and inefficient access to clinical data. User requirements emerged from these themes and primarily focused on information prioritization, data accessibility, and workflow and efficiency.

**Conclusions:**

Notable levels of missing information and inefficient access to clinical data were reported by end users caring for IHT patients at the time of transfer. Conducting user research to understand the current process of IHT, involving users in conceptual design and information architecture, and generating prototypes for feedback from users can aid in designing a solution that meets user needs. The results of these early UCD activities will be used to develop and implement a data platform to support clinicians during IHT.

## Introduction

The transfer of patients between acute care hospitals (interhospital transfer [IHT]) occurs regularly, with one study of nationally representative Medicare data reporting over 100,000 patients undergoing IHT each year and a particularly high frequency of IHT among patients with multiple chronic conditions [1-5]. During IHT, health information exchange (HIE), which can be defined as “appropriate, timely sharing of vital patient information,” is an essential part of the transfer process for appropriate care to be provided to the transferred patient [6]. Importantly, effective HIE encompasses both timely access to data (ie, data exchange), as well as an organized presentation of clinical information (ie, data visualization) so that the receiving or accepting clinician can safely and efficiently provide care to transferred patients without unnecessary cognitive load [7].

Though the concept of HIE is easy to understand, achieving effective HIE has proven difficult due to several factors: (1) accepting clinicians who care for IHT patients after arrival are working within a busy hospital environment. Thus, any solution aimed at improving HIE during IHT must fit seamlessly into the workflow of these busy clinicians, while also not creating delays in patient transfer which could impact patient outcomes; (2) existing interoperability of different electronic health records (EHRs) between different hospital systems is often insufficient, resulting in lack of complete or adequate data exchange [8-13]. In some cases, this is due to active information blocking by developers of certified HIEs or others, but in many cases, it is due to insufficient adoption of interoperability standards [14,15]. Therefore, effective HIE must include adequate interoperability; and (3) even when HIE occurs effectively, there is often a lack of organized presentation of the data; up to 34% of clinicians report delays in their care of transferred patients due to disorganization of medical records requiring extra time to review [16,17]. Thus, improving HIE during IHT must also address the user interface.

There is a critical need for an integrated, interoperable HIE that addresses these 3 factors, along with other domains such as ease of use and usability of the information. At the same time, there is limited research on user requirements to support the development of such HIE.

This is the first phase of a 5-year study that aims to design, implement, and rigorously evaluate the implementation of an HIE platform to improve data access during IHT [18]. This paper reports on the utilization of user-centered design principles to better understand the clinician experience of HIE in the IHT process and identify key user requirements for the design of an effective HIE platform to support IHT. User requirements reflect the user needs of a system and can be used broadly to design solutions to address them.

## Methods

### Design, Setting, Participants, and Recruitment

This study was conducted at Brigham and Women’s Hospital (BWH), Boston, Massachusetts, an 826-bed quaternary care hospital that cares for a high volume of patients who are transferred from other acute care facilities for specialized care. BWH uses Epic (Epic, MyChart, Care Everywhere; Epic Systems Corporation) as their enterprise EHR, which allows the transfer of health information between institutions using Epic through an HIE feature called Care Everywhere [19].

We recruited APPs, direct care attendings, and residents who regularly care for patients who undergo IHT to the general medical, oncology, cardiology, and intensive care unit services at BWH to participate in the design sessions. A general recruitment letter with information describing the goals of the study, human research committee approval, and study team contact information was emailed to all eligible clinicians requesting volunteers to participate in design sessions, with recurring emails sent up to 3 times until target recruitment was reached, ensuring appropriate representation among each participant role. Interested participants could sign up via a link included in the email and were contacted by the study team to coordinate available timing for the design session.

### Ethical Considerations

The study was approved by the Mass General Brigham Institutional Review Board for Human Research (2022P001284). Participants were informed that their participation was voluntary, would not affect their employment, and that all collected data would remain confidential, with results presented only in the aggregate. Participants gave verbal consent to participate in the design sessions. At the completion of the session, participants were given a US $50 gift card for their time.

### Design Sessions

All sessions were conducted virtually over Teams (Microsoft Corp). Sessions were led by a user experience specialist (PG) and assisted by a research team member with content expertise (SM).

**Figure 1.**
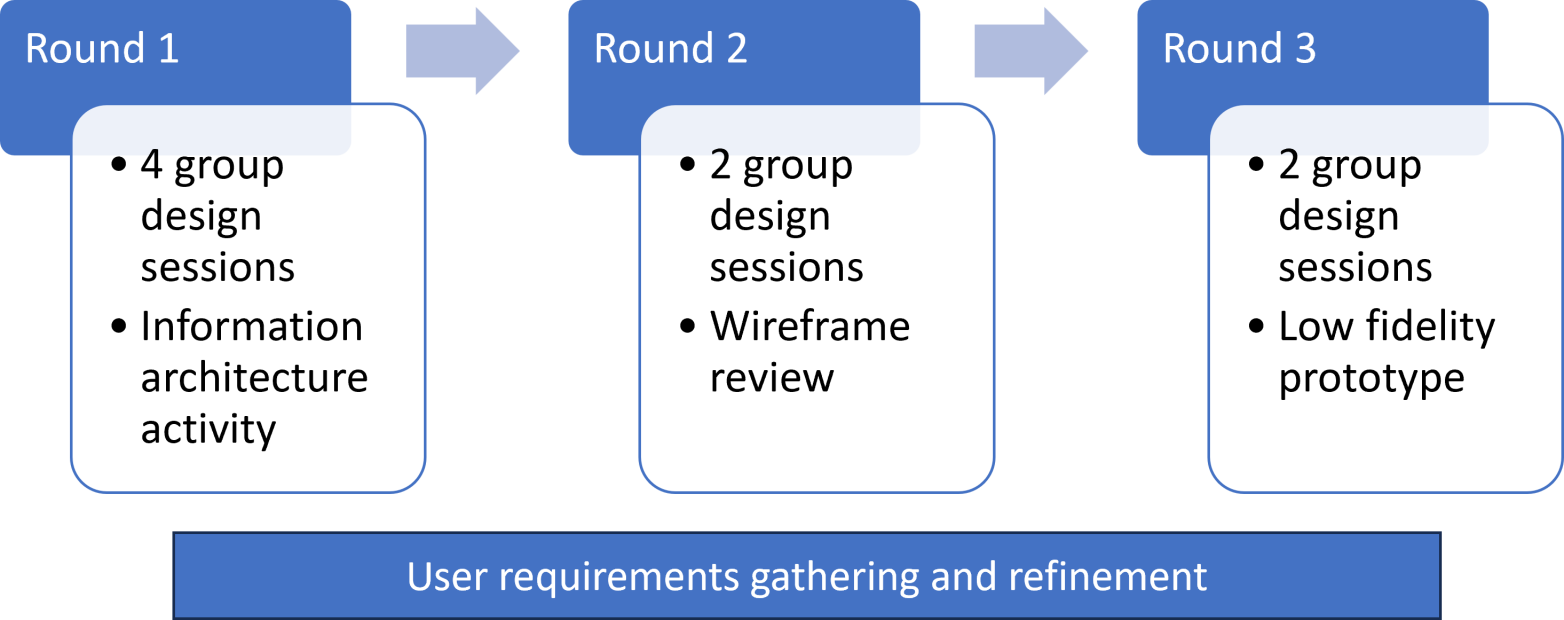
User research process.

Following a user-centered design process (Figure 1), we conducted the first round of 4 group design sessions to understand existing processes around HIE during IHT, using a semistructured group discussion guide (Multimedia Appendix 1). The interview guide was developed by the user experience specialist (PG) and qualitative research expert (RR) with input from the content expert (SM) and additional research team members and piloted prior to participant sessions. The discussion guide covered topics including prompts for sharing the current state of workflow during IHT and interaction with the EHR, with a particular focus on accessing clinical data from hospitals in different systems and with different EHRs, as well as a discussion of barriers and facilitators with the current systems of data access during IHT. During this part of the discussion, we included current screenshots of Epic using redacted real-life patient transfer examples to understand their data-gathering activities more explicitly. Design sessions also gathered participant input on essential clinical data elements available during IHT and included an interactive information architecture activity using previously identified key data elements during IHT (Multimedia Appendix 2), where participants were prompted to group, add, and organize data elements according to their preferences for optimal data review when caring for a transferred patient [20].

Following this first round of design sessions, we performed rapid analyses to generate a preliminary list of user requirements to be refined and discussed during later rounds of design sessions. We drafted an initial wireframe based on these user requirements to be used during our next round of design sessions. The second round of 2 design sessions focused on iteratively refining the draft wireframe and preliminary list of user requirements, with directed input from participants on what they liked and disliked, what was missing, and how they would use, reorganize, group, and manipulate the data. We again performed rapid analysis to develop the draft wireframes into low-fidelity prototypes of an IHT data platform within Epic, developed by Mass General Brigham Digital, which we then presented to end users in the third round of 2 design sessions. In this last stage, we gathered iterative end user feedback to optimize data elements and presentation prior to further development and testing of the IHT data platform within the EHR.

### Analysis

We used a modified framework and rapid analysis approach to analyze the focus group data starting after the first round of design sessions and continuing with each additional session. Sessions were transcribed using the automated feature of Teams, reviewed for accuracy, and then reviewed independently by 2 study team members (JK and PG) to develop a preliminary set of codes. The codebook was refined during multiple meetings among the 2 coders and a third research team member to provide content expertise (SM) until a final codebook was developed. Data were input into MAXQDA qualitative analysis software (VERBI GmbH), and the final analytical framework was applied to participant comments. To further analyze the data, a matrix was created to identify larger themes and subthemes among the data. Data from the information architecture activity was organized using Excel (Microsoft Corp) to identify how often data elements were sorted into any given category.

User requirements emerged from the participant comments, including the information architecture activity. The user requirements reflect the user’s needs, expectations for the system, and what is required to support the user’s goals and tasks. Although we identified solutions to these requirements within our Epic environment, the user needs and expectations are applicable regardless of the platform or technology. The requirements were iterated on throughout the design sessions using a rapid analysis approach and summarized as refinements to the user requirements.

## Results

### Participant Characteristics

Of the 34 clinicians who expressed interest in participating in one of our design sessions during this period of the study, 18 clinicians were selected. We conducted 8 design sessions between February and November 2023, involving 3 medicine residents, 10 APPs, and 5 direct care attendings. Most participants were female (n=14, 78%), general internal medicine clinicians (n=13, 72%), and APPs (n=10, 63%; Table 1).

**Table 1.** Group design session participant demographics.

Characteristics^a^	Participants, n (%)
Age (years)	
<30	3 (17)
30‐35	5 (28)
36‐44	7 (39)
45‐54	3 (17)
Sex	
Female	14 (78)
Male	4 (22)
Race	
Asian	3 (17)
Black or African American	1(6)
White	14 (78)
Ethnicity	
Hispanic	1 (6)
Non-Hispanic	17 (94)
Role	
Advanced practice provider	10 (64)
Direct care attending	5 (23)
Resident	3 (14)
Department	
Cardiology	2 (11)
General or internal medicine	13 (72)
Oncology	2 (11)
Intensive care unit	1 (6)
Years of experience in role	
0‐4	10 (56)
5‐9	5 (28)
10‐15	1 (6)
Over 15	2 (11)
Frequency of IHT^b^ patient admission	
Around once per month	6 (33)
Once every 2‐4 weeks	7 (39)
At least once per week	5 (28)
Time conducting direct care as a responding clinician (%)	
0‐25	3 (17)
26‐50	3 (17)
51‐75	3 (17)
>76	9 (50)

a All characteristics were self-reported, collected via recruitment survey prior to participation.

b IHT: interhospital transfer.

### Design Sessions

#### Summary

The analytic process resulted in multiple codes which were categorized into three main themes and subthemes that reflect the essential aspects of effective HIE during IHT from the perspective of frontline clinicians: (1) communication, (2) data availability and completeness, and (3) efficiency of access to data.

#### Theme 1: Communication

##### Overview

Clinicians tended to frame their responses about data in a way that was often tied to a desire for complementary communication to accompany the discrete patient data available to them within the EHR. Participants expressed that, in current IHT processes, communication is often lacking, and felt as though adding information on the timing of patient arrival or a “warm handoff” (verbal communication to help synthesize the clinical information) would add valuable contextual information to improve their ability to care for IHT patients on arrival. Communication was categorized into 2 subthemes: written and verbal.

##### Written Communication

In addition to specific data elements (as described below), participants also identified a requirement for additional communication related to aspects of the transfer process that would be essential in guiding their workflow. These included transfer process characteristics such as status or timeline of the transfer (eg, final decision to accept and anticipated time of arrival), any other consultants or care team members that were involved in the transfer process (eg, transferring clinician contact information, accepting physician, and consultants involved prior to transfer), and clinical aspects of care that required follow up (eg, pending tests or reports).

##### Verbal Communication

One of the challenges mentioned by accepting clinicians was a lack of verbal communication or “warm handoff.” Participants relayed that with select, complicated patient transfers, the opportunity to have a “warm handoff” was helpful to synthesize the clinical data available to them within the EHR, to help them optimize care for IHT patients. As such, participants identified that the availability of accurate contact information for the transferring physician, as well as the accepting physician, would be beneficial, such that they could then directly contact these individuals to help clarify information as needed. One participant shared, “Patient information can be lost or forgotten due to unclear communication ... if communication for pass off is not addressed, patients can be harmed.”

Notably, there were some overlaps between these 2 subthemes. For example, in many circumstances, participants expressed a need for clearer communication without distinction as to whether they prefer that this occurs via written or verbal communication, particularly related to the contextual aspects of the transfer (eg, timing of arrival).

### Theme 2: Data Availability and Completeness

#### Overview

Participants additionally shared their challenges in being able to access complete data about a patient from the transferring hospital. Data completeness was defined as access to essential clinical patient information to effectively care for the transferred patient on arrival, and was categorized into 6 subthemes: brief summary, imaging, laboratory data, notes, medications, and contact information.

#### Brief Summary

Commonly mentioned as one of the most important data elements that was commonly unavailable to frontline clinicians was a “brief summary” of the clinical scenario. Clinicians described the need for access to a “brief summary” or “snapshot” of the patient, including a “one-liner” on the patient’s active medical issues and pertinent past medical history, the reason for transfer and the patient’s immediate needs, and the patient’s acuity level or illness severity. Participants relayed the need for this summary for all transferred patients and endorsed that this information was especially critical when patients were unable to provide history on their own. Importantly, participants also indicated the need for real-time updates to this summary if the clinical status of the patient were to evolve while awaiting transfer.

#### Radiology Imaging

In addition, one of the most challenging data completeness issues expressed by the participants was the availability of radiology images (eg, the computed tomography scan or chest x-ray images) rather than solely the interpretive reports of the imaging studies completed by the radiologist at the transferring hospital. Participants relayed that EHR system incompatibility, specifically the part of the EHR system that manages radiology images, contributes to the lack of access.

#### Laboratory Data

Participants requested access to specific laboratory data depending on the clinical condition of the patient (eg, cardiac enzymes for patients with cardiac symptoms), as well as information about laboratory tests that were pending at the time of transfer (eg, sent by the transferring hospital but not yet resulted), which was often unavailable at the time of transfer. One participant stated, “…we want to be able to have previous data for what’s happening, is patient getting better or worse.”

#### Clinical Notes

Participants also relayed that incomplete availability of clinical notes felt to be important in caring for transferred patients, including notes related to prior admissions (eg, discharge summaries), prior office visits, and notes related to the patient’s current admission at the transferring hospital (eg, their initial history and physical, or “H & P”). One participant stated their need for “the most recent pertinent data from the hospitalization, if they do not get most of the care from our hospital, we want a bigger time frame of information ... is their health problem new? We need back data or a link out to prior data. That would be helpful.”

#### Medications

Participants relayed challenges with the availability of an accurate medication list at the time of transfer. Importantly, they expressed the desire for access to several different aspects of the patient’s medication list, including an accurate list of medications the patient was taking prior to admission to the transferring hospital to provide historical context, an accurate list of medications the patient was taking at the time of transfer, and particularly a desire for access to the medication administration record, which is a documentation of the timing of medication administration. This latter list was felt essential to be able to make efficient decisions about medication orders when the patient arrives at the hospital.

#### Contact Information

Participants expressed that they infrequently have available to them contact names and numbers they need throughout the transfer process, including the contact information for the transferring clinician (ie, to be able to contact with any questions), as well as the contact information for the patient’s specialists, which in many cases can help provide significant insight into the case.

### Theme 3: Efficiency of Access to Data

#### Overview

In addition to challenges with the availability of data from transferring hospitals, participants noted that, even when available, they experienced inefficient access to it. Efficiency of access to data was categorized into 5 subthemes: dependence on manual processes, EHR organization and presentation, clinical notes, laboratory data and vital signs, and awareness of existing EHR capabilities.

#### Dependence on Manual Processes

Participants explained that in cases when the patient is transferred from a hospital outside the health care system, many processes are manual, such as searching through paper or electronically faxed records that are often PDF scans of paper. They also relayed that occasionally, the clinical data for the patient is on a disc or USB that requires manual uploading and sorting. This dependence on manual processes is felt to be extremely cumbersome and an inefficient way to access patient data.

#### EHR Organization and Presentation

Participants explained that for patients transferring from a hospital within the health care system or from a hospital with the same EHR, the “Care Everywhere” feature of Epic provides access to data but processes of distilling that information and finding relevant data points to the current patient situation is challenging and inefficient. Participants described it as “disorganized, difficult to navigate” and requiring “lots of digging.” In addition, participants describe organizational issues that impact their workflow. For example, one participant shared this about imaging and imaging reports: “Putting images and encounters together would be helpful. They are usually separate from the report.”

#### Clinical Notes

In addition to the incomplete availability of select clinical notes (described above), relevant notes such as the discharge summary and history and physical from the transferring hospital are not always easy to find. In addition, participants shared that, when available, they use the filtering feature for notes and different specialty tabs, such as the cardiology tab, to find relevant notes more quickly.

#### Laboratory Data and Vital Signs

In addition to incomplete access to laboratory data (described above), participants also described frequent inefficiencies in the ability to view historical laboratory data and vital signs to be able to effectively evaluate for trends, particularly if the patient had data in disparate hospital settings or EHRs. Participants expressed a desire for direct access to the most recent laboratory data and vital signs, but with easily accessible historical data organized in a coherent chronological way, even when these data are coming from disparate sources. A participant shared, “I think it’s great to have it [lab data] in one spot. I think not everybody knows how to grab things from Care Everywhere...”

#### Awareness of Existing EHR Capabilities

Most participants noted a lack of familiarity with or use of the “access center note”—a document reflecting a brief summary of the most recent transfer hospital information entered by access center nurses at the time the patient was accepted for transfer until the time of patient arrival. Additionally, most participants relayed not knowing how to locate the “transfer module,” also used by the access center nurses during the acceptance process, which includes a call log and running timeline of transfer hospital information and communication. “Two reasons why people cannot see the transfer module is either because they do not have access to see it or they don’t know what it is (that the information exists) and don’t bother to look for the information in notes.”

### Information Architecture: Organization of Data Elements

In parallel to the above analyses, we analyzed participant feedback from the information architecture activity conducted as part of the first 4 design sessions to identify the preferred organization of essential data elements, naming for the groupings, and additional data elements to include. Participants grouped patient demographic data and transferred hospital contact information together. Participants organized data elements such as the reason for transfer, needs of transferring hospital, and a patient “one-liner” into a “brief summary” or “snapshot” section. Most participants grouped clinical patient data together, including vital signs, laboratory data, microbiology data, radiology data, and other clinical data. Narrative data, like clinical notes and summaries, were grouped together, and medications often got placed in a category of their own.

### User Requirements

Throughout the design sessions, we generated and refined a list of user requirements. The user requirements represent how the system needs to support the work of the end user and were identified from the themes and subthemes outlined above, as well as the results of the information architecture activity. In total, we generated a list of 17 user requirements categorized into information prioritization, data accessibility, and workflow and efficiency:

Information prioritization includes requirements that reflect the user preferences for the order in which they view data (ie, user needs to see select patient data in line-of-sight vs via click-to-expand, eg, second or third priority).

Data accessibility requirements cover the users’ needs for relevant data elements and additional clinical information that is not currently readily accessible to them. For example, users describe a desire to access the various data elements within “medications,” including access to the patient’s home medications (prior to hospitalization at the transferring hospital), medications at the time of transfer, and the medication administration record, which includes details of the timing of most recent medication administration.

Workflow and efficiency include requirements that address the user needs for solutions that work within their current workflow, minimize steps, and provide easier ways to accomplish their task. (ie, user wants flexible displays of data to be able to view the most critical and choose to access more detail).

**Figure 2.**
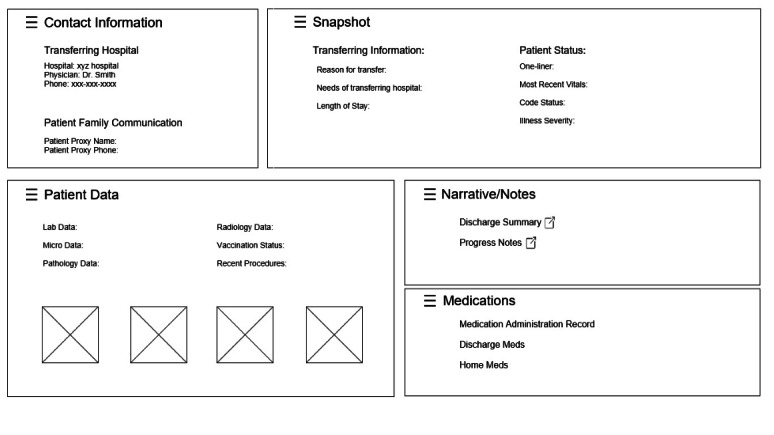
Wireframe of the electronic interhospital data platform.

For each identified user requirement within these 3 categories, we brainstormed potential solutions to address the requirement and iteratively refined each requirement and proposed solution via additional design sessions. Wireframes were developed based on these user requirements and used during subsequent group sessions to elicit feedback and validate requirements (Figure 2). The wireframes included sections for demographic data and transferring hospital contact information; a collated patient “snapshot” or “brief summary” including a “one-liner” and reason for transfer; and sections of clinical data grouped into classical categories including vital signs, laboratory data, microbiology data, radiology data, other clinical data, clinical notes, and medications. Participants from later design sessions shared more specific feedback on how they prefer to view and organize certain data based on refinement from earlier sessions. More details on our user requirements and proposed solutions can be found in Table 2.

**Table 2.** User requirements and proposed design features.

User requirement	Proposed solutions
Information prioritization	
System places contact information as high priority	Suggested data elements: number for transferring floor, name or contact of transferring physician, name of accepting hospital and other clinicians involved, contact information for health care proxy. Contact information section in the top left corner of the summary page.
System places critical clinical information as high priority	Suggested data elements: a brief summary of the patient’s clinical status, reason for transfer, anticipated patient needs in the first 24 hours, length of stay at the transferring hospital, code status, and level of illness severity. “Snapshot” section in the top right corner of the summary page.
System places clinical patient data as mixed high and low priority	Suggested data elements: vitals data, laboratory data, radiology data, microbiology data, pathology data, vaccination status including COVID-19, and recent procedures. Data section in the left center of the summary page. Combination of preview and controls that expand or link out.
System places narrative of clinical course at transferring hospital as mixed higher and lower priority	Suggested data elements: progress notes, discharge or transfer summery, prior discharge summaries, consult notes, history and physical note. Notes section on the right side of the summary page, below the snapshot. Combination of preview and controls that expand or link out.
Data accessibility	
System supports user in efficiently accessing contact information for the patients care team	Provide contact information within line of sight.
System allows easy access to home medications, medications at time of transfer, and medication administration record	Active medication list shown before home medication list. Medications listed in alphabetical order. Access to medication administration record via a link or collapsible content (to reduce overcrowding on the summary page).
System includes a way to view trends in vitals, laboratory data, and historical out of range lab data	Display last 3 values or days. Search function for specific laboratory data. Present an option to view laboratory data in tabular format.
System supports access to current and prior admission notes, progress notes, recent PCP^a^ notes, specialist consult notes, and discharge summary	“Notes” displays patient clinical notes in reverse chronological order. Links to admission notes, progress notes, recent PCP notes, specialist consult notes, and discharge summary.
System allows user to access all prior patient records	Display links to all available outside hospital records.
System allows user to access pending data at time of transfer	Links to pending data included within pertinent sections (eg, laboratory data and microbiology data).
Workflow and efficiency	
System supports user in accessing transferred patient data in one location and in one logical timeline to avoid searching through outside records and faxes	Generate a Patient Summary Page with all outside records pulled into structured sections. List data in reverse chronological order. Provide links to specific data points or outside records for additional information.
System provides flexible displays of data so users can view most critical and choose to access more detail	Provide progressive disclosure for lengthy content. Combination of preview and controls that expand or link out
System provides user with access to the status of a transfer as it evolves during the transfer process	Provide access to the patient timeline documentation as documented by the accepting hospital (generated at time of acceptance for transfer).
System provides user with access to radiology images (not just reports)	Provide access to the radiology image and report within same location. Organize this within the “radiology data” section.
System allows user to choose their timeline for select data presentation	Provide link out to additional historical data.
System allows user to view Patient Summary Page while viewing other windows with detailed information to reduce back and forth between tabs	Design Patient Summary Page as an embedded tab within the electronic health record. Option to open other parts of the patient chart on the side panel while viewing the Patient Summary Page. Option to “pin” page to workbench for easier access.
System allows user to pull data from the Patient Summary Page into their current patient documentation	Provide functionality to pull data from the Patient Summary Page to patient documentation.

a PCP: primary care provider.

## Discussion

### Principal Findings

We identified 3 major themes that are important to address in the design and development of an IHT summary: communication, data completeness, and efficiency of access to data. The user requirements generated from the design sessions were centered around these themes and focused on prioritizing critical information, easily accessing and viewing data from outside sources in one place, and organizing the data within the existing workflow to maximize efficiency. This study highlights the need for one location within the EHR where clinicians can access all the relevant and available data from a transferring hospital as it relates to the care of the transferred patient.

Most prior studies of HIE have focused on the technical requirements for the exchange of data, such as addressing the fragmentation caused by a lack of standards and the governance of EHR vendors [21,22]. Within this framework, several studies have targeted select aspects of HIE; for example, several studies targeted the transfer of medications between systems. One such study relied on qualitative semistructured interviews of key stakeholders to assess challenges and opportunities for more effective information exchange to achieve more accurate medication lists. This study identified similar challenges to our findings, including incomplete patient records and gaps in the availability of digital data, along with barriers related to lack of interoperability and poor IT and change management [23]. Another study reported the use of a design science research methodology, including assessing stakeholder requirements, to develop an interoperable open standards solution for the transfer of medication information between acute and postacute care facilities using 2 different EHRs [24]. Although the transfer of medication information is an essential component of HIE, it does not encompass the entirety of clinical information that should be included with effective HIE. A third study, which also used qualitative semistructured interviews of key stakeholders to address HIE more broadly, identified 3 themes related to user requirements for interoperability of HIE between systems: technological (eg, interoperability challenges), organizational (facilitators of interoperability), and environmental (policy domain) [21]. Other studies have identified similar themes, such as technological and organizational barriers to interoperability [22,25-27]. This study contributes to this body of literature, importantly by highlighting user needs beyond interoperability, particularly regarding the presentation and usability of the data once integrated into the EHR, and the ability to act upon the data within clinical workflow. Our rigorous methodology, using tools such as prototyping and participatory design, ensured that we adequately represented the clinician’s perspective and defined the user requirements [28]. This distinction is essential to ensuring that HIE is effective in allowing data to be easily accessible and understandable by the end user.

While addressing the technical aspects of data availability is critical, our findings suggest that improving clinician experiences during IHT requires more than back-end technical solutions alone. This study found that access to critical data remains a challenge for providers, particularly when it comes to imaging, medication records, and laboratory values. In addition, this study highlights the burden on the clinician when the data is available but difficult to find or digest. Many providers are experiencing significant effort required to “click around” and find the data they need, which has been shown in other studies to contribute to clinician burnout [29]. Even within existing HIE tools that allow access to external data (eg, CareEverywhere within Epic), this study highlights that clinicians still struggle to navigate the information and quickly retrieve essential details needed to care for the patient in front of them. Although EHR vendors are continually improving and working to address these issues, our findings show that there are still significant challenges in distributing these solutions to end users [30]. This may lead to delays in patient care and increased effort for clinicians, negatively impacting the transfer process. This is supported by prior work that demonstrates a high frequency of suboptimal HIE, with up to 35% of patient transfers missing essential clinical data on transfer [16,17,31]. Suboptimal HIE during IHT has also been associated with patient harm, such as therapeutic errors and delays in care, and it may contribute to greater mortality observed among IHT patients with select diagnoses, in addition to other outcomes such as greater resource utilization [4,9,17,32-35].

One significant subtheme that we identified within the data availability and completeness themes was the mention of the “brief summary” or “snapshot” of the patient transfer. This data request was unique in that it is an unstructured piece of information, often evolving and dynamic; therefore, unlike more discrete pieces of data such as lab values or medications, there are challenges in identifying the source and responsibility for this information and making it available to clinicians in an accurate and useful presentation. With advances in existing technologies such as artificial intelligence and natural language processing [36], our findings highlight the potential to leverage these newer modalities to assist in this identified need to synthesize clinical information and present a “brief summary” to the clinician. For example, natural language processing algorithms can be used to analyze unstructured text and parse the data to create a structured patient summary. In addition, artificial intelligence algorithms can pull data from disparate sources and consolidate it into a unified summary for a full picture of a patient’s relevant history.

### Limitations

Our study has several limitations. We gathered requirements from clinicians working at one health care institution using Epic, therefore, the results may not be generalizable to other settings and EHRs. While the user requirements can be applied broadly, the information exchange may vary depending on the health system, so the successful application of these findings may vary depending on the environment. Indeed, any solution that meets these identified user requirements must also comply with data privacy and security regulations, which may restrict the extent of successful information exchange. Future research will include additional sites and EHR systems to understand whether there are additional needs across different health care environments and geographical regions. Furthermore, we restricted the qualitative sessions to direct care attending physicians, residents, and APPs on the medicine, oncology, cardiology, and intensive care units. Thus, the providers who participated may not be representative of all providers. However, we wanted to focus on those who had been most relevant to this study in order to get more detailed feedback about our potential intervention. In exploring solutions to address the user requirements, constraints of the Epic system and compatibility issues surfaced as limitations in addressing the user needs fully. Additional iterative refinement and usability testing will be essential during the development of an HIE platform to ensure its usability and relevance in improving the IHT process, as technology advances and health care culture and workflows evolve.

### Conclusions

Optimal HIE has the potential to improve the safety of IHT and to meet the workflow needs of the clinicians caring for transferred patients. Current EHR interoperability and disorganized, insufficient information make these conditions difficult and can cause delays in patient planning and treatment. We have identified key themes within existing HIE and end user requirements for effective HIE, incorporating both data needs and interface design of key data elements. The next steps include using these key findings to develop an organized IHT HIE platform within the EHR, capable of gathering the most pertinent transfer patient information quickly and securely, with the goal of both bettering patient outcomes and reducing provider burden.

## Supplementary material

10.2196/67884 Multimedia Appendix 1 User research session semistructured discussion guide.

10.2196/67884 Multimedia Appendix 2 Key data elements for interhospital transfer.

## Abbreviations

APP: advanced practice provider
BWH: Brigham and Women’s Hospital
EHR: electronic health record
HIE: health information exchange
IHT: interhospital transfer
