# Supplementary material for: User Requirements and Conceptual Design for an Electronic Data Platform for Interhospital Transfer Between Acute Care Hospitals: User-Centered Design Study
Source: JMIR Hum Factors. 2025 May 30;12:e67884. doi: 10.2196/67884 (PMC12143853; doi:10.2196/67884)
Supplement: Multimedia Appendix 1 [file humanfactors-v12-e67884-s001.docx]

### Part 1: Welcome and Intro (5 mins)

Thank you for participating today. You will be helping us understand the inter-hospital transfer process as part of our study to develop an intervention to improve health information exchange (HIE) during inter-hospital transfer (IHT). Part of the intervention will include an HIE platform accessed by clinicians that would provide clinical information during IHT.

We are interested in everyone’s perspectives and input as part of this discussion. You all were selected to participate as you all have valuable perspectives to provide in thinking through patient transfers. To make this a useful discussion, we encourage and value your honest opinions.

Do you have any questions?

Are you willing to participate in this interview?

We will plan to audio-record this session for future analysis. The recordings and transcripts will only be seen by those people working on this research project and will be used in our analysis.

Do you consent to being recorded?

### Part 2: Discussion of Current State (20 mins)

#### Topics

- Satisfaction with current process during IHT
- Description of workflow re: hospitals in same system, hospitals in different systems with same ehr, hospitals in different systems using different ehrs
  - *Resident prompt:* how does any of this work differ by service (cardiology vs general medicine vs ICU)?
  - *Direct care/hospitalist prompt:* does this work differ by service (general medicine vs oncology)?
  - What works well?
  - What doesn’t work well?
- How do you use the EHR in this process (i.e., where do you look for data?) What data is missing? What works well?
- Who are the key stakeholders in the process?
- Barriers/complications – cases that are more or less difficult?
- What would improve this process?

### Part 3: Discussion of Data Types

- Data Types (15 mins):
  - Share data types
  - Group activity: Move them around, add, remove, group, prioritize
- Design Features (20 mins):
  - ‘Yes and’ activity
    - For example, Participant 1: “the summary should allow me to see trends of their data” Participant 2 “Yes and…..”
  - What do you need to do with the data?
  - What data presentations do you find helpful?
  - What challenges do you foresee?
  - When in workflow are certain data/features important?
  - What are the most important features?

### Part 4: Wrap-Up

- Is there anything else we should be thinking about for this intervention that we haven’t discussed?
- Would you like to be engaged in this project in the future?
