# Supplementary material for: User Requirements and Conceptual Design for an Electronic Data Platform for Interhospital Transfer Between Acute Care Hospitals: User-Centered Design Study
Source: JMIR Hum Factors. 2025 May 30;12:e67884. doi: 10.2196/67884 (PMC12143853; doi:10.2196/67884)
Supplement: Multimedia Appendix 2 [file humanfactors-v12-e67884-s002.docx]

Key Data Elements for IHT

| - **^†^Selection of illness severity as “unstable,” “watcher,” “stable” (manual)** |
| --- |
| - **^ǂ^Reason for transfer (manual)** - **Discharge/Transfer summary (auto-populate)** - **^§^Progress notes (auto-populate)** - **^§^Medication administration record (MAR) (auto-populate)** - **Vital signs (current, trend) (auto-populate)** - **Laboratory data (current, trend) (auto-populate)** - **Microbiology data (auto-populate)** - **Radiology imaging + report (auto-populate)** - **Pathology data (auto-populate)** - **COVID status (auto-populate)** - **Code status (auto-populate)** |
| - **^¥^Anticipated needs in first 24 hours (manual)** |
| - **Name of transferring hospital (auto-populate)** - **Contact number of transferring floor (auto-populate)** - **Name/contact number for healthcare proxy (HCP) (auto-populate)** |
| - **Name/contact of transferring physician (to contact as needed) (auto-populate)** |

^†^Illness Severity: Unstable= Current clinical status (i.e., vital signs, vital sign trend, or laboratory data) indicates this patient is in need of or at high-risk of requiring intensive care unit (ICU); Watcher=Current clinical status indicates this patient is at moderate-risk of requiring ICU AND/OR the patient was in the ICU at the transferring hospital within the past 24 hours; Stable=Current clinical status indicates this patient is at low-risk of requiring ICU.

^ǂ^Reason for transfer: Higher Level Care/Need for specialized care or procedure; Patient/family preference; Continuity of care; Capacity issues at transferring hospital; Other

^§^Recent progress notes and MAR were determined important by frontline stakeholders to allow quick review of current clinical status and medications as they are often available for viewing prior to the transfer summary, which can take time to complete/transfer

^¥^Anticipated needs: Nothing anticipated; Consult service (specify:__); Imaging (specify:__); Procedure (specify:__); Other (specify:__)

Mueller S, Murray M, Goralnick E, Kelly C, Fiskio JM, Yoon C, et al. Implementation of a standardised accept note to improve communication during inter-hospital transfer: a prospective cohort study. BMJ Open Qual. 2023;12(4). doi:10.1136/bmjoq-2023-002518
